# Linking Experimental Models to Pathophysiology: Oxidative Stress and DNA Damage in Cardiovascular Diseases

**DOI:** 10.3390/ijms27093931

**Published:** 2026-04-28

**Authors:** Shahin Gavanji, Hazem Zaki, Priyadarshini Panjwani, Eman M. Othman

**Affiliations:** 1 Department of Plant Biotechnology, Medicinal Plants Research Centre, Isfahan (Khorasgan) Branch, Islamic Azad University, Isfahan 81744-73441, Iran; gavanji.shahin2@gmail.com; 2 Faculty of Pharmacy, Deraya University, Minia 61111, Egypt; hazemzaki770@gmail.com; 3 Nonclinical Development Unit, CSL Innovation GmbH, 35041 Marburg, Germany; priya.panjwani@cslbehring.com; 4 Department of Biochemistry, Faculty of Pharmacy, Minia University, Minia 61519, Egypt; 5 Cancer Therapy Research Center (CTRC), Department of Biochemistry-I, Biocenter, University of Wuerzburg, 97074 Wuerzburg, Germany

**Keywords:** cardiovascular, oxidative stress, in vitro, in-vivo, assay, ROS

## Abstract

There has been an immense concern in the healthcare industry about the globally raising rate of cardiovascular disease (CVD). As per recent WHO reports, CVD is the leading cause of disability, hospitalization and premature death. Studies indicate that oxidative stress negatively impacts the heart and vascular system, which could potentially lead to myocardial infarction, hypertension, cardiomyopathies, atherosclerosis and diabetic heart failure, highlighting its significance as a prognostic indicator in cardiovascular conditions. Nowadays, many common experimental assays are used for in-vitro and in-vivo evaluation of oxidative stress and its negative effects on the cardiovascular system. This review aims to serve as a comprehensive guide for researchers seeking to evaluate the impact of oxidative stress on DNA damage in CVD utilizing standardized methods published by leading institutions. To achieve this, we analyzed 208 relevant articles from prominent databases such as Scopus, PubMed, ScienceDirect, etc., summarizing experimental validation of oxidative stress measurements from 1955 to the present. Oxidative stress-induced DNA damage is a key driver of cardiovascular disease progression, yet experimental approaches to study it remain highly variable. This review systematically summarizes established in-vitro and in-vivo models, oxidative stress inducers, and analytical assays used in cardiovascular research. By integrating mechanistic insights with standardized methodologies, it provides a practical framework to guide model selection, improve reproducibility, and enhance translational relevance. This work serves as a concise reference for researchers investigating redox biology, cardiovascular pathology, and antioxidant-based therapeutic strategies.

## 1. Introduction

Medical experts around the world are increasingly concerned about the rising rate of cardiovascular disease (CVD). It is the leading cause of early death and disability and puts huge strain on healthcare systems and economies. In fact, CVD is considered the most expensive disease, with an estimated indirect cost of USD 237 billion each year [1,2].

Research shows that many common risk factors like smoking, drinking alcohol, lack of physical activity, poor diet, hormonal changes due to stress, lack of proper sleep, obesity, and hypertension can lead to CVD [3]. These factors are known to increase oxidative stress in the body [4,5,6]. When the body produces too many reactive oxygen species (ROS) and does not have enough antioxidants to balance them out, it leads to oxidative stress. This can damage important molecules like DNA, lipids, and proteins [7,8], which contributes to many diseases including cancers, metabolic disorders, and hormonal conditions leading to CVD [9,10]. Oxidative stress negatively impacts the heart and blood vessels and can lead to conditions such as myocardial infarction, atherosclerosis, and diabetic heart failure [11,12]. The main sources of ROS in the heart include enzymes such as xanthine oxidoreductase, monoamine oxidases (MAO), NADPH oxidases (NOXes), mitochondria, cytochromes P450 (CYP), and nitric oxide (NO) synthases [7,9]. Tracking oxidative stress in blood, serum, and plasma has helped researchers identify biomarkers that play key roles in the development of heart diseases. These markers play significant roles in coronary artery disease (CAD) development and can also be used to predict the disease progression of CVD [13]. For example, serum lipid hydroperoxides (LOOHs) are primary products of fatty acid peroxidation, while malondialdehyde (MDA) a stable end product of lipid peroxidation (LP) results from an interaction between radical species and PUFAs [14]. Elevated levels of LOOH and MDA have been associated with cardiovascular risk factors like smoking and diabetes mellitus, therefore highlighting their utility to predict primary and secondary CVD [15,16]. Currently various in-vitro and in-vivo assays are employed to assess oxidative stress and its detrimental impact on CVD. These standard testing methods are instrumental not only in evaluating CVD risk but also in screening for novel drugs with antioxidant activity. Over the past decade, several studies focused on the development and validation of such assays, offering practical tools to study and access oxidative stress in CVD and to design novel antioxidant-based therapeutic strategies. Our review provides comprehensive guidance for researchers by summarizing widely adopted and standardized assays used globally across academic, institutional, preclinical, and clinical research settings.

## 2. Methods

**Figure 1:**
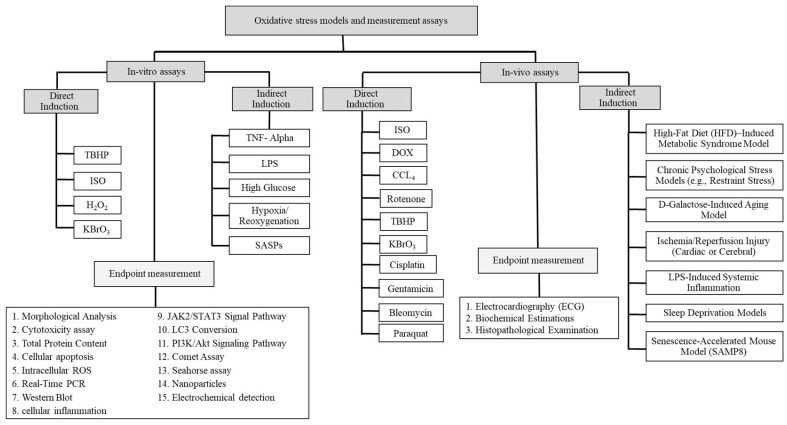
Methods for in-vitro and in-vivo oxidative stress induction and evaluation.

To put together this review article, we explored a wide range of publications, scientific protocols, and books available on major research databases Web of Science, PubMed, Scopus, Google Scholar, and Science Direct. We focused on keywords such as oxidative stress assays and ROS in cardiovascular diseases, in-vitro and in-vivo studies, and methods to assess oxidative stress and DNA damage in CVD. We used two sets of criteria to select the source; the first was the broad filter that included in-vitro and in-vivo methods for measuring oxidative stress. The second was more detailed focusing on studies specifically testing oxidative stress in CVD and heart failure. Our comprehensive search includes publications dating back to 1955 to ensure that we captured both the foundational work and recent advancements (Figure 1).

## 3. Experimental In-Vitro Models

One of the key steps in preclinical research on CVD is evaluating the detrimental effects of oxidative stress on the heart and vascular system. Oxidative stress can lead to various cellular abnormalities that ultimately contribute to cardiac dysfunction. To study these effects, it is important to select an appropriate in-vitro model that allows precise control over experimental conditions [17]. In-vitro models including primary or induced pluripotent single cardiac cells, 2D and 3D cultures, tissue culture, and microfluidic platforms are essential tools. They support researchers in exploring the molecular mechanisms of ROS, along with its interaction with cells, and gather valuable insights before moving on to in-vivo assays and de-risk major in-vivo studies by contributing to 3R (Table 1). A critical factor for obtaining reliable results is selecting the right cell lines [18]. The choice depends on several factors, including the available laboratory equipment, type of in-vitro models, functional characteristics of the cells, and the specific goals of the assay [17,18]. A variety of cell lines are commonly used in CVD, including cell lines like AT-1 [19], HL-1 [20], AC [21], and H9c2 [22]; primary neonatal and adult cardiomyocytes [23,24] and stem or progenitor cells [25]. Among these, the H9c2 cell line has been most frequently used in CVD [22,26,27]. Derived from BD1X rat heart tissue, the H9c2 cell line exhibits several properties of skeletal muscle and offers a versatile platform to investigate oxidative damage and its role in cardiac systems [28].

## 4. Direct Inducers of Oxidative Stress In-Vitro Model

The H9c2 myocardial cell line is widely considered an ideal model for studying oxidative stress and DNA damage in cardiovascular research. The most common inducers are summarized in Table 2.

### 4.1. Tert-Butyl Hydroperoxide (TBHP) in In-Vitro Model

TBHP is one of the most commonly used compounds for investigating induction of oxidative stress in in-vitro models. It is well-known for its ability to trigger damage and apoptosis in cardiomyocyte [52,53]. To perform this assay, H9c2 cells are cultured in Dulbecco’s modified Eagle medium (DMEM) supplemented with 10% fetal bovine serum (FBS), 100 μg/mL streptomycin, 100 U/mL penicillin, 1.5 g/L sodium bicarbonate, and 4 mM L-glutamine. The cells are maintained in a humidified incubator at 37 °C with 5% CO_2_ and 95% O_2_. After 2–3 passages of the stock cultures, cells are seeded into 96-well plates at a density of 5.0 × 10^3^ cells per well. Once attached, cells are treated with 150–200 μM TBHP for 1 h, depending on the readout planned for the endpoint measurement. This provides a consistent and reliable setup for the investigation of oxidative stress antioxidant strategies [42,54].

### 4.2. Isoproterenol (ISO)

ISO-induced myocardial ischemia is a classical model used to evaluate the cardio-protective effects of various pharmacological agents. ISO causes severe oxidative stress in the myocardium, leading to infarct-like necrosis of the myocardium.

To perform this assay, the H9c2 cells are cultured and divided into several groups: negative control, ISO-treated (50 μmol/L), positive control (0.1 mmol/L captopril), and treatment groups with different concentrations of the test item with predicted antioxidant properties. All groups are incubated for 24 h. Afterwards, all groups except the negative control group are treated with 50 μmol/L ISO and incubated for 48 h. Depending on the endpoint, read-out is conducted and analyzed [55,56,57].

### 4.3. Hydrogen Peroxide (H_2_O_2_)

H_2_O_2_ is a commonly used ROS inducer for generating oxidative stress and cellular damage in various in-vitro models [58]. Endogenous H_2_O_2_ in immune and vascular smooth muscle cells contributes to the generation of oxidative stress which leads to endothelial dysfunction and development of various vascular diseases [50]. To perform this assay, seed H9c2 cells in 96-well plates at a density of 5 × 10^3^ cells per well, allowing them to reach the logarithmic growth phase. Then, divide them into different groups and treat them with 200–400 μM of H_2_O_2_ for 24 h. Upon treatment, cells can be used for subsequent experiments [51].

### 4.4. Potassium Bromate (KBrO_3_) in In-Vitro Model

KBrO_3_ is a food additive that has commonly been used in the production of drinking water disinfected with ozone [59]. Due to its strong oxidizing properties, KBrO_3_ can act as an inducer of oxidative stress leading to lipid peroxidation and DNA damage [60]. The free radicals generated by KBrO_3_ are known cardiac toxins, and the heart is very sensitive to its effect [61,62].

To perform this assay, seed the H9c2 cells in 96-well plates at a density of 5 × 10^3^ cells per well and allow them to reach the logarithmic growth phase. Then, divide them into different groups and treat them with 250 μM KBrO_3_. Incubate the cells in a humidified incubator at 37 °C with 5% CO_2_ and 95% O_2_ for 72 h [63].

To ensure accurate interpretation of experiments described in Section 4.1, Section 4.2, Section 4.3 and Section 4.4, it is essential to complement the induction protocols with clearly defined biochemical and cellular readouts. Successful induction of oxidative stress by agents such as TBHP, ISO, H_2_O_2_, or KBrO_3_ should be confirmed through a combination of assays reflecting redox imbalance and cellular damage. Key indicators include a significant increase in intracellular reactive oxygen species (ROS), commonly measured using fluorescent probes (e.g., DCFDA), along with a concomitant depletion of endogenous antioxidant defenses such as reduced glutathione (GSH) and superoxide dismutase (SOD) activity. In parallel, elevated levels of lipid peroxidation products, particularly malondialdehyde (MDA), serve as reliable markers of oxidative membrane damage. Functional outcomes such as reduced cell viability (e.g., MTT assay), morphological alterations, and increased apoptosis (e.g., Annexin-V staining, caspase activation) further validate the oxidative insult. At the molecular level, oxidative stress is typically associated with upregulation of pro-apoptotic genes (e.g., Bax, Caspase-3, Caspase-9) and downregulation of anti-apoptotic markers such as Bcl-2, as well as modulation of stress-responsive signaling pathways including JAK2/STAT3 and PI3K/Akt. Importantly, interpretation of these results should always be performed relative to appropriate controls (untreated, inducer-treated, and intervention groups), and consideration should be given to dose- and time-dependent effects. Integrating these endpoints allows for robust validation of oxidative stress induction and ensures reproducibility and biological relevance of the experimental findings.

## 5. Indirect Inducers of Oxidative Stress in In-Vitro Model

### 5.1. Tumor Necrosis Factor-Alpha (TNF-α)

TNF-α is a pro-inflammatory cytokine that promotes oxidative stress via activation of mitochondrial dysfunction and NADPH oxidase pathways. It plays a central role in the enhancement of ROS induction in various cardiac and vascular models.

To perform this assay, H9c2 cells are cultured and seeded into 96-well plates at a density of 5 × 10^3^ cells per well. Once attached, cells are treated with 10–50 ng/mL TNF-α for 24 h. The resultant oxidative stress can be measured through ROS-specific fluorescent probes and antioxidant enzyme activity assays [64].

### 5.2. Lipopolysaccharide (LPS)

LPS, a structural component of Gram-negative bacterial walls, is a widely used stimulator of inflammation-induced oxidative stress. Upon binding to TLR4, LPS activates transcription factors like NF-κB, which enhances intracellular ROS generation and impairs mitochondrial integrity.

To model this in-vitro, H9c2 cells are treated with 1 μg/mL LPS for 24–48 h under standard conditions. The LPS model is particularly useful in evaluating antioxidant properties of natural and synthetic compounds [65].

### 5.3. High Glucose (HG)

Chronic exposure to high glucose mimics diabetic hyperglycemia and induces oxidative stress through enhanced mitochondrial ROS production and suppression of antioxidant pathways. This model is frequently used in diabetic cardiomyopathy research.

To perform this assay, H9c2 cells are cultured with 25 or 33 mM D-glucose for 48–72 h. The cells are then analyzed for markers of oxidative damage such as increased ROS, lipid peroxidation, and changes in GSH levels [66].

### 5.4. Hypoxia/Reoxygenation (H/R)

The hypoxia/reoxygenation model simulates ischemia-reperfusion injury in-vitro, where the sudden influx of oxygen leads to a burst of ROS formation, especially from mitochondria.

For this assay, H9c2 cells are exposed to hypoxic conditions (1% O_2_) for 4–6 h using a hypoxia chamber or gas control incubator, followed by reoxygenation in normoxic conditions (21% O_2_) for 2–24 h. This model closely mimics clinical scenarios such as myocardial infarction or stroke [67].

### 5.5. Senescent Cell Co-Culture

Senescent cells are metabolically active and exhibit a senescence-associated secretory phenotype (SASP), which includes pro-oxidant cytokines and matrix-degrading enzymes. This microenvironment leads to increased oxidative burden in neighboring cells.

To simulate this, H9c2 cells can either be co-cultured with senescent fibroblasts or treated with 0.2–0.5 μM doxorubicin for 24 h to induce senescence. After 48–72 h of exposure, oxidative stress parameters can be assessed [68].

## 6. Morphological Analysis for Oxidative Stress and DNA Damage in Cardiovascular Diseases

The H9c2 myocardial cell line is a well-established model for in-vitro evaluation of oxidative stress. To examine morphological changes, the cells should be fixed and stained following the YF^®^488-labeled phalloidin staining protocol.

Briefly, fix several cells on ice using 4% paraformaldehyde solution for 15 min. Then wash gently with PBS. Permeabilize using 0.5% Triton X-100 in PBS at room temperature for 10 min, followed by another PBS wash. Dilute 5 μL of YF^®^488-labeled Phalloidin stock solution in 200 μL of PBS and incubate cells with this mixture for 30 min at RT. After staining, wash off extra dye with PBS. Finally, observe the morphological changes using a 400× inverted fluorescence microscope. For analysis, randomly select four to six views from each sample and measure the cell surface with the available software such as Oplenic software [57,69,70,71].

## 7. Cytotoxicity Assay

### 7.1. MTT Cell Viability Assay

To assess cell viability, the MTT assay is commonly used. Briefly, pre-incubate H9C2 suspension cells in DMEM within 96-well plates at a concentration of 5 × 10^4^ cells/100 μL/well. Incubate overnight in a humidified incubator at 37 °C with 5% CO_2_. The next day, replace the culture medium with fresh medium containing different groups—such as negative controls, positive controls, and treatment groups. Cells are treated with different concentrations of oxidative stress inducers and vehicle controls. Incubate the cells under the same conditions for 6 h. Post treatment, add 10 μL of 10% MTT solution to each well and incubate for 4 h at 37 °C. Once incubation is complete, remove the supernatants and dissolve the formazan crystals in 100 μL of solubilization solution. Measure the absorbance by microplate reader at 630 nm [72]. To calculate the percentage of viable cells, use the following equation:

$$\mathrm{Cell}\mathrm{viability}(\%)=[\frac{{Mean\;OD}_{sample}}{{Mean\;OD}_{blank}}]\times 100.$$

### 7.2. Determination of Total Protein Content

To assess total protein content, along with SOD, GSH, and MDA levels use the appropriate commercial test kit. For this assay, centrifuge different treatment groups, such as negative control, positive control, and treatment groups. Treat cells with different concentrations of oxidative stress inducers and vehicle controls. Post incubation, centrifuge at 137× *g*, and collect the cell pellet. Add 0.3 mL of normal saline to each pellet for the total protein, GSH, and SOD assays. For MDA assay, add 0.5 mL TBA working solution and follow the manufacturer’s instructions for accurate detection [73,74,75].

### 7.3. Determination of Cellular Apoptosis

To assess cellular apoptosis, begin by seeding cells at a concentration of 1 × 10^6^. Wash the cells with phosphate-buffered saline (PBS) and centrifuge cells at 200× *g* for 5 min. Resuspend the resulting pellet in 100 μL of Annexin-V-FLUOS labeling solution and incubate the cells at 15–25 °C for 15 min. After incubation, use a fluorescence microscope to analyze apoptosis. For the detection Annexin-V-FLUOS staining kit can be used [76].

In addition to staining, quantitative real-time PCR (qRT-PCR) can be used to evaluate apoptosis at the gene expression level. This involves measuring the mRNA expression of key apoptotic markers including Bcl-2, Bcl-2/Bax, Bax, Caspase-3, Caspase-8, and Caspase-9. Studies have shown that oxidative stress inducers promote apoptosis in cells, raising the expression of pro-apoptotic genes such as Bax, Caspase-3, Caspase-8, and Caspase-9 while reducing expression of anti-apoptotic genes like Bcl-2 and reducing the Bcl-2/Bax ratio [77,78,79,80].

### 7.4. Determination of Intracellular ROS

One of the key aspects of cellular behavior is its ability to respond to ROS within the intracellular environment. The level of intracellular ROS can be measured by commercial ROS detection kits.

To perform this assay, harvest H9c2 cells and wash them thrice to remove traces of residual medium. Then add serum-free DMEM to cells. Stain them with 0.5 μL of 100 mmol/L of 2′,7′–dichlorofluorescin diacetate (DCFDA), a cell permeable fluorescent probe, and place the cells in a dark room for 30 min. The intracellular ROS level is then measured by flow cytometric evaluation of fluorescence intensity of DCFDA in cells [57,69,81].

## 8. Quantitative Real-Time PCR

To perform this assay, extract total RNA from H9c2 cells using an RNA extraction kit. Then synthesize cDNA using a cDNA synthesis kit. The mRNA expression levels of ANP, BNP, β-MHC, IL-6, TNF-α, Bax, Bcl-2, Caspase-3, Caspase-8, and Caspase-9 are then quantified using real-time PCR [47,82].

Changes in the expression of genes such as ANP, BNP, β-MHC, IL-6, TNF-α, Bax, Bcl-2, and Caspases are closely associated with the pathophysiological mechanisms underlying cardiovascular disease. For instance, ANP and BNP are well-established markers of cardiac stress and are significantly upregulated during myocardial hypertrophy and heart failure, reflecting increased wall tension and ventricular dysfunction. Similarly, β-MHC expression is elevated in response to pathological cardiac remodeling and is indicative of a shift toward a fetal gene program commonly observed in diseased myocardium. Pro-inflammatory cytokines such as IL-6 and TNF-α play a central role in mediating inflammation-driven cardiac injury, contributing to endothelial dysfunction, fibrosis, and progression of heart failure. In parallel, oxidative stress-induced apoptosis is a key contributor to cardiomyocyte loss, where increased expression of pro-apoptotic genes such as Bax and Caspases (Caspase-3, -8, and -9), along with decreased expression of the anti-apoptotic gene Bcl-2, promotes cell death and exacerbates cardiac dysfunction. Collectively, these gene expression changes not only serve as molecular indicators of oxidative damage but also provide mechanistic insight into disease progression, making them critical endpoints for evaluating cardiovascular injury and therapeutic interventions [47,82].

## 9. Western Blot

For the Western blot assay, culture H9C2 cells (5 × 10^6^ cells) for 24 h, and prepare several treatment groups including positive and negative controls. After treatment, extract total protein and determine concentration using a BCA protein assay kit. Separate proteins by sodium dodecyl sulphate polyacrylamide gel electrophoresis (SDS-PAGE) and transfer them to polyvinylidene fluoride (PVDF) membranes. Block the membranes with skim milk and incubate overnight with primary antibodies against p-JAK2, JAK2, p-STAT3, STAT3, TNF-α, Caspase-3, PI3K, Akt, mTOR, LC3-II, LC3-I, and GAPDH. Finally, analyze band intensities with digital tools to evaluate variation in protein levels [47,83]. The protein expression changes detected by Western blot analysis provide critical mechanistic insight into the progression of cardiovascular disease under oxidative stress conditions. Key signaling molecules such as JAK2, STAT3, PI3K, Akt, and mTOR are central regulators of cardiomyocyte survival, inflammation, hypertrophy, and metabolic adaptation. Under oxidative stress, aberrant activation or suppression of these pathways reflects pathological remodeling processes. For example, increased phosphorylation of JAK2 and STAT3 is commonly associated with inflammatory signaling and cardiac hypertrophy, while dysregulation of the PI3K/Akt/mTOR axis can impair cell survival, promote apoptosis, and alter autophagic balance in cardiomyocytes. Elevated levels of pro-inflammatory proteins such as TNF-α further indicate activation of inflammatory cascades that contribute to endothelial dysfunction and myocardial injury. Additionally, increased expression of apoptotic markers such as Caspase-3 confirms activation of programmed cell death pathways, which plays a key role in cardiomyocyte loss during disease progression. Monitoring these protein level alterations, therefore, not only allows validation of oxidative stress induction but also provides functional insight into downstream pathological events, making Western blot analysis a crucial tool for linking molecular signaling changes to cardiovascular outcomes [47,83].

## 10. Measurement of Cellular Inflammation

To assess inflammation, quantitative real-time PCR to measure cellular mRNA expression levels of IL-6 and TNF-α was widely reported. Several studies demonstrate that expression of both IL-6 and TNF-α increases in cells under oxidative stress. In addition, protein expression of TNF-α also rises in ROS-treated groups. Therefore, this assay setup is used to evaluate the impact of ROS on IL-6 and TNF-α expression to measure anti-inflammatory effects of tested compounds [83,84,85].

## 11. Evaluation of JAK2/STAT3 Signal Pathway

Several studies demonstrated that oxidative stress inducers can induce cardiomyocyte hypertrophy (CH). To perform this assay, evaluate the protein expression levels of JAK2, p-JAK2, STAT3, and p-STAT3 in the different treatment groups.

Oxidative stress caused by increased levels of ROS has been shown to promote cardiac hypertrophy (add reference). To evaluate this, protein expressions of JAK2, p-JAK2, STAT3 and p-STAT3 have been measured in appropriate cellular systems [86,87,88].

## 12. Evaluation of LC3 Conversion

LC3 (microtubule-associated protein 1A/1B-light chain 3) protein plays a central role in autophagy, the process by which cells break down and recycle damaged components to maintain equilibrium. LC3 is a key autophagosome marker and plays an important role in the final stages of autophagosome formation. LC3-I is an inactive form of protein floating in the cytoplasm. LC3-I gets converted to LC3-II, which attaches to the membrane of the autophagosome. The LC3-II/LC3-I ratio is commonly used to measure autophagy levels. LC3-II to LC3-I conversion can be detected with Western blot [89,90,91].

The LC3-II to LC3-I ratio is widely used as a quantitative indicator of autophagy; however, its interpretation requires careful consideration. In general, an increase in the LC3-II/LC3-I ratio reflects enhanced autophagosome formation and is indicative of autophagy activation, which is commonly observed in cardiomyocytes under oxidative stress conditions. Conversely, a low or unchanged LC3-II/LC3-I ratio suggests basal or absent autophagic activity. It is important to note that accumulation of LC3-II alone does not always indicate increased autophagic flux, as it may also result from impaired degradation of autophagosomes due to lysosomal dysfunction. Therefore, to accurately determine whether autophagy is truly induced or inhibited, the LC3-II/LC3-I ratio should be interpreted alongside additional markers such as p62/SQSTM1 degradation or assessed in the presence of lysosomal inhibitors (e.g., bafilomycin A1 or chloroquine). In the context of cardiovascular disease, moderate increases in autophagy are often considered protective by removing damaged cellular components, whereas excessive or impaired autophagy may contribute to cardiomyocyte death and disease progression [89,90,91].

## 13. PI3K/Akt Signaling Pathway

The PI3K/Akt/mTOR signaling pathway is a key regulator for cell growth, survival, metabolism, and autophagy, all of which are sensitive to increases in ROS levels. This pathway was very often reported in experimental models to investigate cellular response to oxidative stress and should be investigated. Western blot analysis to assess levels of PI3K, Akt, and mTOR post-treatment was performed. Reduction in levels of p-AKT and p-mTOR was indicative of oxidative stress in treated cells [92,93].

To ensure clear interpretation of the PI3K/Akt/mTOR pathway under oxidative stress conditions, it is important to distinguish between protein expression and pathway activity. In the present context, the pathway was primarily evaluated through downstream phosphorylation events, where reduced levels of phosphorylated Akt (p-Akt) and mTOR (p-mTOR) indicate suppression of PI3K/Akt/mTOR signaling. However, total PI3K protein levels were not directly assessed, and therefore, no definitive conclusion can be drawn regarding changes in PI3K expression itself. This distinction is critical, as oxidative stress may impair signaling activity without necessarily altering total protein abundance. Accordingly, the observed effects should be interpreted as a decrease in pathway activity rather than a confirmed reduction in PI3K protein levels.

## 14. Comet Assay to Evaluate Oxidative DNA Damage

Several methods exist to assess oxidative DNA damage in mammalian cells under in- vitro conditions. Among them is the comet assay, which is a simple and precise technique for quantifying DNA damage.

To perform this assay, seed the cells and mix them with 180 μL of 0.5% low-melting agarose. Spread the mix onto fully frosted slides that have already been coated with a layer of 1% normal-melting agarose. Next, incubate slides in lysis solution composed of 0.1 M EDTA, 10 g/L N-lauroylsarcosine sodium salt (pH 10), 2.5 M NaCl, and 0.01 M Tris, supplemented with 10% dimethyl sulfoxide (DMSO) and 1% Triton X-100 for 1 h at 4 °C. After lysis, place them in the electrophoresis solution for 20 min. Then, perform electrophoresis at 25 V and 300 Ma for 20 min. In the next step, neutralize the slides using 0.4 M Tris buffer and dehydrate them in the cold methanol at −20 °C for 10 min. After dehydration, place the slides in the incubator at 37 °C to dry and then store them at RT. Finally, stain the DNA with Gel Red/ DABCO (diazabicyclo-octane) solution and analyze the images from each slide (50 cells per sample). The main evaluation parameter in this assay is the percentage of DNA tails which reflects the extent of oxidative damage [94].

## 15. Seahorse Assay

The Seahorse XFp Analyzer is widely used and is one of the most powerful tools for analyzing and evaluating cellular respiration. This assay allows measurement of the oxygen consumption rate (OCR) and extracellular acidification rate (ECAR), which provides valuable information about metabolic dysfunction, mitochondrial function, and oxidative stress in viable cells [95]. One of the key advantages of this assay compared to traditional oxygraph methods is its requirement for a low number of cells, which is particularly useful for metabolic analysis in diseases associated with mitochondrial dysfunction; hence it is one of the most commercially used techniques in metabolism research [96].

To perform this assay, the Seahorse XF24 Extracellular Flux Analyzer (Agilent, CA, USA) and its dedicated software could be used. First, seed 2 × 10^4^ H9c2 cells per well in XF24 cell culture plates. Incubate cells in XF assay medium, which is a modified DMEM medium, at 37 °C for 1 h. Next, cells are exposed to FCCP (a respiratory uncoupler) to measure maximal respiratory capacity of the cell; oligomycin, an ATP synthase inhibitor, to assess ATP-linked respiration, and rotenone plus antimycin A, inhibitors of complex I and III, to quantify non-mitochondrial respiration. The results obtained after such analysis allow detailed profiling of mitochondrial function, which includes basal respiration, ATP production, proton leak, maximal respiration, and spare respiratory capacity. These parameters are essential for evaluating the impact of oxidative stress and potential protective or therapeutic interventions on cellular metabolism. The main advantage of using Seahorse XFp Analyzer is the fact that it analyzes live cells, allowing compounds to be tested in a dose-dependent manner. This enables precise determination of their effects on cellular respiration and facilitates the calculation of lethal doses. [97,98].

## 16. Nanoparticles for Detection and Monitoring the Reactive Oxygen Species (ROS)

In recent years, a growing number of research studies have focused on developing innovative tools for ROS detection, tracking, and monitoring. To meet this need, various types of nanosensors and nanomaterials have been designed to detect early signs of oxidative stress within biological systems [99] (Table 3). Numerous studies have shown that fluorescent nanomaterials, when combined with nanocarriers, can significantly improve the optical performance of fluorescent probes by minimizing photobleaching and enhancing signal stability [100,101,102]. The integration of nanomaterials in ROS detection offers several advantages, including low toxicity, improved solubility, and precise customization of fluorescent imaging probes for specific biological environments [103]. Furthermore, the incorporation of nanomaterials into biosensors can enhance the physiochemical properties of these analytical devices, improving their sensitivity, specificity, and biocompatibility with in-vivo applications [104]. One of the emerging tools for evaluating ROS at tissue depth is photoacoustic (PA) imaging, a hybrid method that combines optical contrast with ultrasound resolution. PA imaging provides high spatial resolution and deep tissue penetration, making it a promising tool for ROS detection in live organisms [105,106]. Notably, photoacoustic sensors have been successfully used in cardiovascular research to monitor oxidative stress [107,108]. A study by Jung et al. (2018) introduced thrombus-specific theranostic (T-FBM) nanoparticles which significantly enhance H_2_O_2_-triggered photoacoustic (PA) signals and demonstrated antithrombotic effects of these molecules [109].

## 17. Electrochemical Detection

Electrochemical methods have emerged as precise, sensitive and straightforward ways to measure the ROS under both, in-vitro and in-vivo conditions. In these methods, the platinized nanoelectrode is commonly used as ROS detector due to its high sensitivity and rapid response time. Electrochemical techniques are especially valuable in biological assessments, where they are applied to evaluate drug-induced ROS production. These methods are not only easy to use but also offer detection sensitivity, short incubation time, and the ability to detect low levels of intracellular ROS with high spatial resolution [138]. Advancements in nanotechnology have further enhanced the capabilities of these tools. The development of these nanoelectrodes has enabled single-cell electrochemical monitoring and analysis, opening new possibilities for assessing oxidative stress at cellular and subcellular levels [139,140,141,142,143]. These ultra-fine nanoelectrodes can penetrate the cell membrane and directly measure ROS levels within cellular organelles such as the nucleus and mitochondria [141,143,144]. A notable example of this application was reported by Actis and colleagues in 2014, where they used a carbon fiber disk-shaped nanoelectrode to measure ROS levels in melanoma cells demonstrating the potential of this technique for real-time intracellular ROS detection and tracking [145]. Further research has led to the development of quartz nanopipette-based nanoelectrode, which offer enhanced precision compared to first-generation nanoelectrodes in intracellular ROS measurement. These electrodes provide unique advantages in single-cell analysis, enabling researchers to measure oxidative stress triggers at the individual cell level and its subcellular compartments, which will contribute immensely to the study of cancer and disease models [146,147].

## 18. Electron Paramagnetic (Spin) Resonance (EPR/ESR)

The development of sensitive, specific and precise assays for ROS detection is essential for advancing our understanding of oxidative stress and its trigger in disease pathology [148,149]. One of the most widely recognized and reliable techniques for detecting free radicals is spin trapping [150,151]. It is a well-established technique for the detection of short-lived free radicals which otherwise are too reactive, unstable, and transient to observe or measure directly. In this technique, these fleeting radicals react with spin traps—a special chemical moiety that forms stable covalent bonds with free radicals resulting in the production of persistent nitroxide molecules that can be detected and measured using electron paramagnetic resonance (EPR), also known as electron spin resonance (ESR) [152]. This method is based on the principle of microwave radiation absorption by unpaired electrons in a magnetic field. Key components of the EPR system include a microwave generator and a resonator cavity, which work together to detect the magnetic signals generated by spin-labelled radicals, leading to detection of ROS [153]. Among the most effective probes used in this method are cyclic hydroxylamines, which are highly reactive with ROS. These probes are converted into stable nitroxide radicals, making them ideal for quantitative EPR analysis. Several factors enhance the efficiency of hydroxylamine-based spin probes in ROS detection including their reactivity, accumulation rate, membrane permeability, organelle-targeting ability, and chemical stability [154,155,156]. Due to enhanced specificity and ability to detect real-time radical formation, EPR remains one of the gold-standard techniques for studying oxidative stress in both in-vitro and in-vivo systems.

## 19. Extracellular H_2_O_2_ Detection by Amplex Red

The Amplex Red assay is a widely used and reliable tool for the detection of extracellular H_2_O_2_ in biological samples [157]. This assay is based on the oxidation of Amplex Red reagent (10-acetyl-3,7-dihydroxyphenoxazine) in the presence of horseradish peroxidase (HRP), producing highly fluorescent and stable compound resorufin. To perform the assay, transfer three aortic segments (approximately 2 mm in length) into a 96-well plate. Add 10 μmol/L of Amplex Red with 0.2 U/mL of HRP, then incubate the samples in a dark place at 37 °C for 1 h using Krebs Ringer’s phosphate glucose buffer (KRPG). After incubation, separate the buffer from the tissue, and measure fluorescence at 530 nm excitation (with an appropriate emission filter around 590 nm) [153,158]. This assay is highly sensitive, allowing for quantitative measurement of low concentrations of extracellular H_2_O_2_. However, it is important to protect the reaction from light and to optimize HRP concentration to avoid non-specific background signals.

## 20. Genetic Sensors for Mitochondrial ROS Measurement

Genetically encoded fluorescent biosensors have become precise and efficient tools to assess redox signaling and ROS dynamics within live cells. These protein-based sensors can be specifically targeted to cellular sub-compartments, such as mitochondria, making them invaluable for studying localized ROS production, particularly mitochondrial H_2_O_2_ release [159,160]. Recent advances in biosensor technology have led to the design of engineered probes that specifically and reliably detect H_2_O_2_. These probes offer key advantages such as high sensitivity, specificity reversibility, and the ability to enable real-time detection and visualization of redox flux under a variety of physiological and pathological conditions. Two of the most widely used genetically encoded H_2_O_2_ sensors are roGFP2-Orp1 and HyPer [161,162,163]. RoGFP2-Orp1is a redox-sensitive green fluorescent protein (roGFP) fused with the Orp1 peroxidase domain, enabling selective H_2_O_2_ detection through reversible oxidation, whereas HyPer is a sensor derived from a permuted yellow fluorescent protein (cpYFP) fused to the bacterial H_2_O_2_-sensing protein OxyR. It provides dynamic and ratiometric detection of intracellular H_2_O_2_ [163,164,165]. Both biosensors have been successfully studied in various cell types, including neurons, cardiomyocytes, and immune cells, allowing researchers to understand how ROS fluctuate in response to stimuli, drugs, or stress conditions. Their ability to monitor real-time oxidative events makes them a powerful complement to traditional chemical probes [160,166,167].

## 21. In-Vivo Assays

### 21.1. Experimental In-Vivo Models

While in-silico and in-vitro models provide valuable insights into heart function in an isolated system outside the influence of physiological factors in a controlled experimental setting, they cannot replicate the complexity of a living organism. Therefore, in- vivo models remain essential for accurately studying the cardiovascular system and understanding key mechanisms of oxidative stress and DNA damage in a physiological context [168,169,170,171].

The effects of ROS induction in-vivo can be directly modeled using a variety of chemical agents that either trigger free radicals’ generation or disrupt mitochondrial and antioxidant defense systems. These compounds can lead to oxidative stress in a specific organ, and, in many cases, systemic effects are observed, leading to an animal model that can be utilized to study a specific scientific question.

### 21.2. Direct Inducers of Oxidative Stress In-Vivo

Oxidative stress can be experimentally triggered in an animal using chemical agents such as isoproterenol (ISO), a synthetic β-adrenergic agonist that mimics the effects of excessive sympathetic stimulation. Numerous studies have shown that ISO administration increases heart weight-to-body weight ratio and elevates serum levels of cardiac injury markers such as aspartate aminotransferase (AST), creatine kinase-MB isoenzyme (CK-MB), lactate dehydrogenase (LDH), malondialdehyde (MDA), cardiac troponin I (cTnI), and creatine phosphokinase (CPK). It also leads to ST-segment elevation, reduced R-wave amplitude, QT interval prolongation, and increased heart rate. In addition, it leads to structural damage like edema and myocardial necrosis, which can be observed via histology. This model is commonly used to study the cardioprotective effects of antioxidant compounds and ISO-induced myocardial infarction (MI) in both in-vitro and in-vivo settings [172,173]. To perform the study, the animals are divided into three major groups: a vehicle–control group, an ISO group receiving 85 mg/kg subcutaneously, and treatment groups receiving the test compound prior to ISO administration. The treatment group is used to evaluate the potential protective or therapeutic effects of the test compound against ISO-induced myocardial injury. ISO is typically administered for two consecutive days, and all animals are sacrificed or assessed 12 h after the final dose to analyze biochemical, histological, and functional parameters. One of the major drawbacks of such a model is intense myocardial damage without involving vascular occlusion or chronic remodeling processes. Additionally, its outcomes can vary significantly with dose, strain, and systemic effects, limiting its translational relevance.

### 21.3. Doxorubicin (DOX)

Doxorubicin is an anthracycline chemotherapeutic agent known to induce dose-dependent cardiotoxicity. Its redox cycling within mitochondria results in the generation of superoxide and hydroxyl radicals, contributing to lipid peroxidation and mitochondrial damage. Repeated administration of DOX leads to progressive cardiac dysfunction, mimicking chronic heart failure.

In rodent models, DOX is administered intraperitoneally at cumulative doses of 10–20 mg/kg over 2–3 weeks. The protocol may involve a single high dose or multiple spaced doses to simulate chronic injury. Myocardium is particularly vulnerable to DOX, as it targets mitochondrial function. Animals show reduced ejection fraction, increased oxidative markers, and fibrotic remodeling in cardiac tissue [174,175].

### 21.4. Carbon Tetrachloride (CCl_4_)

CCl_4_ is a potent hepatotoxin, but it also induces systemic oxidative stress, including in cardiac and renal tissues. It is metabolized by cytochrome P450 enzymes into trichloromethyl radicals that initiate lipid peroxidation.

In animal studies, CCl_4_ is typically diluted 1:1 in olive oil and administered at 1–2 mL/kg intraperitoneally. The oxidative burden can be assessed in the liver, heart, and serum through MDA levels, antioxidant enzyme activity, and histology [176,177]. Inflammatory mediators such as TNF-α, IL-1β, and NF-κB activation lead to spillover from CCl_4_ hepatotoxicity and damage the myocardium in chronic conditions.

### 21.5. Cisplatin

Cisplatin is a platinum-based chemotherapeutic agent widely used to induce nephrotoxicity in animal models. It impairs mitochondrial respiration, generates ROS, and depletes intracellular glutathione, particularly in renal tubular cells. It affects cardiac function but impacts cardio-renal crosstalk. Cisplatin is administered intraperitoneally at 5–10 mg/kg. Animals develop acute kidney injury, characterized by elevated serum creatinine, BUN, renal lipid peroxidation, and tubular necrosis [178,179].

### 21.6. Gentamicin

Gentamicin, an aminoglycoside antibiotic, causes oxidative damage primarily in the kidneys by accumulating in proximal tubules and generating mitochondrial superoxide. In rats, daily intraperitoneal administration of 80–120 mg/kg for 5–10 days induces nephrotoxicity, confirmed by raised creatinine, urea, and histological damage. Prolonged exposure to gentamicin leads to oxidative stress in the kidneys, which increases lipid peroxidation and ROS. Histology of these animals would reveal cardiomyocyte vacuolization, edema, and focal necrosis. Some reports demonstrated decreased heart rate and contractility [180,181].

### 21.7. Bleomycin

Bleomycin is used to simulate oxidative lung injury and pulmonary fibrosis. Upon administration, it generates iron-catalyzed ROS that cause DNA strand breakage and release of inflammatory cytokines, which induces oxidative stress in cardiomyocytes indirectly.

The model involves a single intratracheal or intraperitoneal dose of 2–3 mg/kg. Within 7–14 days, animals exhibit impaired lung function, increased collagen deposition, and histological features of fibrosis [182,183].

### 21.8. Rotenone

Rotenone is a mitochondrial complex I inhibitor that impairs oxidative phosphorylation and increases intracellular ROS, especially in dopaminergic neurons. It is frequently used to model Parkinson’s disease in-vivo. Rotenone exposure upregulates oxidative stress response genes in the heart and elevates oxidative damage in heart mitochondria in rodent models, even when damage is subtle histologically. Animals receive chronic subcutaneous doses of 1–3 mg/kg for 4–6 weeks. Behavioral assessments reveal motor impairment, while brain tissue analysis confirms dopaminergic loss and oxidative damage [184,185].

### 21.9. Paraquat

Paraquat is a herbicide known for being a mitochondria-targeted redox cycler that interacts with complex I which generates ROS and leads to acute cardiac stress. Chronic exposure leads to metabolic programing and contractile defects in rodent models. Tempol pre-treatment partially reverses cardiac signatures, making it a good mechanism to probe. In rodents, a single intraperitoneal dose of 10–20 mg/kg results in significant ROS generation, pulmonary edema, and myocardial necrosis upon long exposure [186,187].

### 21.10. Tert-Butyl Hydroperoxide (TBHP)in In-Vivo Model

TBHP is a synthetic organic peroxide that undergoes heme-catalyzed decomposition, leading to alkoxyl and peroxyl radical formation. These molecules directly cause lipid peroxidation and oxidative DNA damage. Cardiomyocytes are especially sensitive to TBHP due to high mitochondrial density and dependence on oxidative metabolism. TBHP is used to study acute oxidative stress, particularly in liver and kidney models. It is a good model to study mechanistic redox studies but is less translatable as compared to DOX or ISO models.

A single intraperitoneal dose of 2–5 mmol/kg leads to measurable oxidative injury within a few hours, often evident as increased MDA levels, reduced GSH, and histological damage [188,189].

### 21.11. Potassium Bromate (KBrO_3_) in In-Vivo Model

KBrO_3_ is a strong oxidizing agent that generates reactive oxygen species and causes oxidative DNA damage, particularly in renal tissue. The signs of oxidative stress can be observed histologically in the heart even though the kidney is the classical target organ. In experimental models, oral or intraperitoneal administration of 100–200 mg/kg induces glomerular injury, proteinuria, and elevated lipid peroxidation markers [190].

### 21.12. Indirect Inducers of Oxidative Stress in In-Vivo Model

Oxidative stress can also develop indirectly in experimental animal models due to metabolic, inflammatory, psychological, or age-related imbalances that shift the redox system toward pro-oxidant dominance. These models do not rely on direct chemical pro-oxidants but instead trigger endogenous ROS production as a secondary effect of the biological process. They are increasingly used in translational research for diseases where oxidative stress is not the primary insult but a secondary consequence of systemic dysregulation [191].

### 21.13. High-Fat Diet (HFD)-Induced Metabolic Stress

The rodent model of a high-fat diet (HFD) is commonly used to investigate oxidative stress resulting from metabolic syndrome. Chronic intake of saturated fats leads to insulin resistance, adipocyte hypertrophy, and mitochondrial dysfunction. This causes excessive ROS generation, mainly in the liver, adipose tissue, and heart. Cardiac tissue is often impacted irreversibly due to high dependency on mitochondria. To generate the model, animals maintained on a 60% fat diet for 12–16 weeks exhibit increased levels of malondialdehyde (MDA), 4-hydroxynonenal (4-HNE), and reduced antioxidant enzyme activity in metabolic tissues. To evaluate therapeutic mechanisms, animals are split into two groups: a control group receiving normal chow and an HFD-fed group. Blood and tissues are collected at designated endpoints to assess oxidative damage and inflammation. Although the model recapitulates many features of human metabolic disease, inter-strain variability and the long duration required to establish significant pathology can be limitations [192,193].

### 21.14. Lipopolysaccharide (LPS)-Induced Systemic Inflammation

Lipopolysaccharide (LPS), a bacterial endotoxin, is widely used to initiate oxidative stress via systemic inflammation. LPS activates toll-like receptor 4 (TLR4) in immune cells, leading to cytokine release, nitric oxide production, and ROS generation through NADPH oxidase. Elevated plasma levels of TNF-α, IL-6, nitric oxide, and protein carbonyls are commonly observed after LPS injection. To generate the model, animals are divided into a control group and an LPS group receiving a single intraperitoneal dose of 5 mg/kg. Tissues such as liver, lung, and brain are collected 6–24 h post-administration for biochemical and histological evaluation. The LPS model is ideal for screening anti-inflammatory and antioxidant compounds under acute immune challenge. However, the short-lived nature of the response limits its relevance for chronic disease modeling [194].

### 21.15. D-Galactose-Induced Aging Model

D-galactose, when administered over extended periods, accelerates aging by promoting oxidative stress via the formation of advanced glycation end-products and mitochondrial dysfunction. Rats or mice receiving 100–150 mg/kg/day subcutaneously for 6–8 weeks show increased lipid peroxidation, protein oxidation, and decreased superoxide dismutase (SOD) activity in the brain, liver, and kidneys. The model is generated by assigning animals to control and D-galactose-treated groups. Behavioral assessments are often performed in parallel to examine cognitive effects, followed by tissue collection for oxidative biomarkers. This model is widely used in anti-aging and neurodegenerative studies. One drawback is that it mimics accelerated aging rather than natural senescence, which may not fully reflect chronic aging mechanisms.

### 21.16. Hypoxia/Reoxygenation (H/R) Injury Model

Hypoxia followed by reoxygenation replicates ischemia-reperfusion injury, where a burst of ROS is generated upon oxygen restoration. This is primarily driven by mitochondrial electron transport chain instability, xanthine oxidase activation, and neutrophil infiltration. In myocardial or cerebral H/R models, oxidative stress is accompanied by elevated MDA, reduced GSH, and mitochondrial swelling. The cytokine response that follows the initial hypoxic insult often shapes the extent and prognosis of the injury [195]. To induce this model, rodents are subjected to controlled hypoxia (e.g., via coronary artery ligation or hypoxia chambers) for 30–60 min, followed by normoxic reoxygenation for 2–24 h. Sham-operated animals serve as controls. This model effectively mirrors clinical scenarios such as myocardial infarction or stroke but requires surgical expertise and standardization [67,196].

### 21.17. Chronic Restraint Stress Model

Prolonged psychological stress is a recognized inducer of oxidative stress in the brain and cardiovascular system. It activates the hypothalamic–pituitary–adrenal axis, elevating glucocorticoid levels and altering redox signaling. Chronic restraint stress increases brain ROS levels, disrupts mitochondrial membrane potential, and decreases SOD and catalase activity in regions like the hippocampus. To induce the model, rodents are placed in plastic restrainers for 4–6 h per day over 14–21 days. A control group is handled similarly without restraint. After the exposure period, behavioral assessments are followed by tissue analysis for oxidative markers. While highly relevant to stress-related disorders, variability in animal temperament and environmental sensitivity can affect reproducibility [197].

### 21.18. Sleep Deprivation Model

Sleep deprivation is associated with increased oxidative stress, especially in the central nervous system. Disrupted sleep leads to enhanced ROS production, impaired antioxidant defenses, and neuronal damage, particularly in memory-related regions like the hippocampus.

This model is implemented using gentle handling or rotating platform systems to prevent REM or total sleep for 24–72 h. Following deprivation, animals show elevated brain MDA, decreased GSH, and impaired performance in behavioral tests. Control animals are housed under similar lighting and temperature conditions without intervention. Despite being non-invasive, the model demands continuous monitoring and often shows variability in stress levels induced by the setup itself [198].

### 21.19. Senescence-Accelerated Mouse Model (SAMP8)

The senescence-accelerated mouse prone 8 (SAMP8) strain is a spontaneous aging model that naturally exhibits increased oxidative stress, cognitive decline, and mitochondrial dysfunction at an early age. These mice show elevated brain MDA levels, increased protein oxidation, and reduced antioxidant enzyme expression by 5–6 months of age.

Unlike other models, no intervention is needed. Age-matched SAMR1 mice are used as controls. Tissues from brain, heart, and skeletal muscle are typically collected for biochemical and histological analysis. SAMP8 is well-established in aging research and provides a stable platform for long-term studies. However, genetic drift and the cost of colony maintenance can be limiting factors [199,200].

## 22. In-Vivo Assays for Oxidative Stress and DNA Damage in Cardiovascular Diseases

### 22.1. Non-Invasive and Translational Imaging Modalities

Electrocardiography (ECG): ECG is for real-time assessment of cardiac structure and function (e.g., ejection fraction, wall motion abnormalities) in oxidative stress models.To perform ECG recordings, Sprague–Dawley SD rats are anesthetized and positioned supine approximately for 30 min. Next, acupuncture needle electrodes are inserted subcutaneously based on lead II configuration, i.e., right foreleg, left foreleg, and left rear leg. ECG signal and heart rate are recorded at every 1 min interval every 5 min using, for example, a PowerLab system (ADInstruments, Colorado Springs, CO, USA) and data are analyzed by LabChart 7 software [173]; special attention should be given to changes in the ST-segment, which can indicate ischemia or myocardial injury.

Cardiac magnetic resonance imaging (MRI): for high-resolution evaluation of myocardial fibrosis, edema, and functional parameters [201].

ROS-specific in-vivo probes: including fluorescent and photoacoustic probes such as lanthanide-ion nanoparticles (GdVO Eu): These nanoparticles allow for real-time, quantitative monitoring of ROS kinetics in inflammation models through photoluminescence modulation.

Genetically Encoded Sensors (e.g., HyPer, RoGFP): These protein-based sensors enable, for example, visualization in live zebrafish, offering highly spatial-resolved monitoring of wound healing.

Cy-DNBS (Near-Infrared): A robust probe for dynamically monitoring biothiols, which are indicators of redox status, both in-vitro and in-vivo, which enables real-time visualization and quantification of oxidative stress in living organisms [202,203,204].

### 22.2. Biochemical Estimations

After blood collection, centrifuge and separate the serum. Next, the following biomarkers should be measured by using a commercially available kit, these are routinely used in laboratory settings: troponin I (cTnI), aspartate aminotransferase (AST), creatine phosphokinase (CPK), creatine kinase-MB isoenzyme (CK-MB), lactate dehydrogenase (LDH), and malondialdehyde (MDA). These should be measures, as they can be indicative of myocardial injury and oxidative damage [205,206,207,208].

### 22.3. Histopathological Examination

For histopathological assessment, after rats are sacrificed in accordance with ethical guidelines and their hearts are excised, immediately fix them in 10% buffered formalin. The ventricular mass is sectioned longitudinally from the apex to the base, followed by routine dehydration with graded alcohol and clearing with xylene before embedding in paraffin wax. Thin histological sections of approximately 5 µm are then prepared by slicing the paraffin block using a microtome. These sections are then stained with hematoxylin and eosin (H & E) and examined under a light microscope to evaluate changes in tissue architecture. Key structural and pathological changes such as edema, cellular infiltration, and myocardial necrosis should be evaluated to understand the extent of myocardial damage [209,210,211]. Special stains can be performed on the consecutive paraffin sections to characterize the cellular subtypes in case of massive infiltration and restructuring. This examination plays a crucial role in assessing oxidative stress-induced myocardial damage, as oxidative injury often manifests histologically through features such as interstitial edema, inflammatory cell infiltration, and myocardial fiber necrosis. These structural changes are consistent with the downstream effects of reactive oxygen species (ROS) on cardiac tissue integrity and are commonly observed in oxidative stress-related models of myocardial injury.

## 23. Critical Comparison of Experimental Models and Guidance for Model Selection

While a wide range of in-vitro and in-vivo models are available to study oxidative stress and DNA damage in cardiovascular diseases, their applicability varies significantly depending on the research objective. A major challenge in the field is balancing experimental control, reproducibility, and translational relevance, as no single model fully captures all aspects of human cardiovascular pathology.

### 23.1. Reliability and Reproducibility of In-Vitro Models

In-vitro systems provide highly controlled environments, making them particularly valuable for mechanistic investigations. Among these, immortalized cell lines such as H9c2 are widely used due to their ease of culture, cost-effectiveness, and relatively high reproducibility across laboratories. Chemical inducers such as H_2_O_2_ and TBHP enable precise and rapid induction of oxidative stress, resulting in consistent and quantifiable outcomes. These features make such systems highly reliable for studying intracellular signalling pathways, redox imbalance, and early-stage cellular responses. However, these models have inherent limitations. Immortalized cell lines may not fully recapitulate the phenotype of primary cardiomyocytes, and their responses to oxidative stress or pharmacological agents can differ from native tissue. Additionally, two-dimensional (2D) cultures fail to reproduce the structural and mechanical complexity of cardiac tissue, which may affect the interpretation of functional outcomes.

Advanced systems such as three-dimensional cultures (3D), co-culture models, and microfluidic platforms offer improved physiological relevance by incorporating cell–cell interactions and tissue-like architecture. Despite this, they often suffer from lower reproducibility due to increased technical complexity and variability in fabrication and handling protocols.

### 23.2. Reliability and Translational Value of In-Vivo Models

In-vivo models remain essential for capturing the systemic and multifactorial nature of cardiovascular diseases. Direct chemical induction models, such as isoproterenol (ISO) and doxorubicin (DOX), are widely used due to their ability to generate robust and reproducible oxidative stress phenotypes within a short time frame. These models are particularly suitable for screening cardioprotective agents and studying acute injury mechanisms. However, their translational relevance is limited by the fact that they often induce exaggerated or non-physiological damage. For example, ISO-induced myocardial injury lacks features such as vascular occlusion and chronic remodelling, which are central to human myocardial infarction.

Indirect models, including high-fat diet (HFD), lipopolysaccharide (LPS), and aging-related systems, better reflect the complex pathophysiology of human disease. These models incorporate metabolic, inflammatory, and environmental components that contribute to oxidative stress in clinical settings. As a result, they offer greater translational relevance. Nevertheless, they are associated with increased variability, longer experimental timelines, and sensitivity to factors such as animal strain, diet composition, and environmental conditions.

### 23.3. Comparative Assessment of Analytical Methods

A similar trade-off exists among analytical techniques used to measure oxidative stress. Fluorescent probes such as DCFDA are widely adopted due to their simplicity and suitability for high-throughput screening; however, they lack specificity and may produce artifacts. In contrast, electron paramagnetic resonance (EPR) is considered a gold-standard method for direct detection of free radicals, offering high specificity and sensitivity, albeit with lower accessibility and throughput.

Functional assays such as Seahorse analysis provide indirect but highly informative insights into mitochondrial function and metabolic alterations associated with oxidative stress. Electrochemical and nanoparticle-based sensors offer emerging advantages in sensitivity and spatial resolution, though their application is still evolving.

### 23.4. Model Selection Based on Research Objectives

The choice of model should be guided by the specific research questions:

For mechanistic studies, highly controlled in-vitro systems using defined oxidative stress inducers (e.g., H_2_O_2_, TBHP) are most appropriate due to their reproducibility and ease of manipulation. In the case of drug screening and cardioprotective evaluation, reproducible in-vivo models such as ISO or DOX-induced injury provide standardized and quantifiable endpoints, and for studies related to disease progression and clinical translation, indirect models such as HFD, aging models, or hypoxia/reoxygenation systems offer greater physiological relevance despite increased variability (Table 4)

## 24. Conclusions and Future Perspective

Oxidative stress plays a central role in the development and progression of many diseases yet studying it in a lab setting is not straightforward. Over the years, healthcare professionals have developed a wide variety of in-vitro and in-vivo models to replicate oxidative conditions, each with its own strengths and weaknesses. Direct chemical inducers like TBHP, ISO, or H_2_O_2_ offer quick and controlled induction of oxidative stress but often lack the complexity seen in actual disease states, which is influenced by multiple factors. On the other hand, indirect models like high-fat diets, LPS exposure, or aging-based setups better reflect physiological conditions, but the results can often vary depending on the dosage, exposure, and strain background. What is often missing from literature is a clear, side-by-side comparison of these models in one place. This review aims to fill that gap by compiling a detailed yet accessible overview of the most widely used oxidative stress models. It includes not only how these models are set up, but also what to expect in terms of biomarkers, tissue responses, and practical challenges. The addition of redox proteomics insights and models like the SAMP8 mouse helps connect molecular mechanisms to disease progression. By bringing together both direct and indirect inducers, this paper offers a practical toolkit for scientists designing studies in cardiovascular, metabolic, or neurodegenerative contexts. It encourages more thoughtful model selection based on research questions rather than convenience alone. In doing so, it bridges the gap between simplified systems and complex diseases. Ultimately, this review is meant to serve as a reference point—one that helps researchers navigate the crowded and sometimes confusing space of oxidative stress modeling.

## Figures and Tables

**Table 1:** In-vitro models for the assessment of oxidative stress in the cardiovascular system.

In-Vitro *Models*	Advantages	Disadvantages
**Cardiac single cell**	Provide information about characteristics of each cardiac cell (20) Help us to quantify the various mechanical properties in individual cells [29] It can help to evaluate the electrophysiology of asingle cardiomyocytes (CMs) [30]	The structure and lattice-like network misalignment of myofibrils can limit their efficiency of contractile activity modeling [31] There are cell-to-cell differences and variations in the cell size, myofibril alignment myocyte type, shape which cause the incorrect measurements in the result [31] The isolated cardiomyocytes have different responses to drugs in cells and groups and can lead to different results [17].
**Two-dimensional (2D) cell cultures**	This method has a closer condition to the in-vivo state [32] It helps us to assess the molecular signaling pathways [32] Useable for cardiotoxicity evaluations and gene therapy approaches [33,34]	Sometimes has variations in cell morphology and polarity [35] The cellular and extracellular environments have problems in interaction (30) May offer misleading results [35]
**Three- dimensional (3D) cell cultures**	Express a wide range of proteins in the CMs culture in comparison with 2D cultures [36]	The materials which have been used in the construction of 3D cardiac cultures can reduce cell survival rates and increase the thickness [37] Geometric design limitation [17]
**Coculture**	Simple [17,38] It provides information to study the cell–cell interactions [39]	The continuous cell lines affection which sometimes show phenotypes that have not observed in the primary cells [40]
**Microfluidic cell culture**	It helps us to have cellular behavior information Provide a platform to study the physiology of normal heart cells and help to create the disease for therapeutic evaluation [35] It provides precise control over cell culture [39]	Sometimes accompanying cell cultures with microfluidic devices can face to problem [41]

**Table 2:** Common induction of oxidative stress in cardiac cell culture (H9c2).

Inducers	Experimental Observation	Molecular Mechanisms	Inducer Dose	EC50/Appotosis
**TBHP**	Downregulate the Bcl2 as an anti-apoptotic protein and upregulate the Bax protein as an apoptotic protein [42] It can reduce the cellular antioxidant enzymes in the cell [42] Induce cell death [42] Rhodamine123 fluorescence will decrease by 66% [42]	Provided by cytochrome P450, which can lead to generation of peroxyl (LOO○) and alkoxyl (LO○) radicals, can initiate the lipoperoxidation (LPO) of membrane phospholipids with devastating reactions [43] Depletion of GSH by oxidation to its disulphide form (GSSG) [44] It can express the 21 genes that are involved in ROS [45]	150 μM [42]	>150 µM [46]
**ISO**	The cells are obviously hypertrophic [47] Increase the mRNA expression levels of ANP, β-MHC, and BNP [47] It decreases the level of GSH and SOD and increases the MDA [47] Increase the level of IL-6 and TNF-α [47]	It causes an imbalance of antioxidants and oxidants in the myocardium that can lead to myocardial injury [48,49] It enhances the phosphorylation levels of STAT3 and JAK2 [47]	50 μmol∙L^−1^ [47]	50–300 µM [50]
**H_2_O_2_**	It induces hypertrophy in cells by affecting the ERK1/2 and Akt signaling pathways [51]	It can induce the PI3K/Akt signaling pathway [51]	200–400 µM [51]	471.8 ± 27.5 µM [51]
**KBrO_3_**	The cell size will increase at concentrations of <250 μM [44]	Increase the expression of two cardiac hypertrophy markers, including β-Myosin Heavy Chain (β-MHC) and the brain/B-type natriuretic peptides (BNP) [44]	<250 μM [44]	>300 µM [44]

**Table 3:** Application of nanomaterials in ROS measurement.

Nano Sensors Nanomicelles/Nanopolymer/Carbon Nanotubes/Metallic	Applications	Advantages	Disadvantages	References
**Luminescence (e.g., quantum dots or lanthanide-doped nanoparticles)**	Detection of nM ROS and H_2_O_2_ for intracellular and in-vivo models	Highly favorable for tissue imaging by near-infrared ROS measurement in Photodynamic therapy	Unspecific	[110,111,112,113,114]
**Fluorescent-quenching (e.g., gold nanoparticles or graphene oxide-based systems)**	Detection of intracellular nM ROS	No sign of Photobleaching Precise and stable	Metallic nanoparticles induced cytotoxicity	[115,116,117,118,119]
**Surface-enhanced Raman spectroscopy (SERS) (e.g., silver or gold nanoparticles)**	Evaluation of Intracellular redox potential	Stable Reversible	Exhibits the pH-sensitivity Raman microscopy equipment required Raman microscopy equipment, complicated assay design	[120,121]
**ROS-dye encapsulation (e.g., Hydrocyanine-conjugated nanoparticles)**	Detection nM and μM of ROS Assessment of tumor associated ROS	Higher signal and sensitivity Efficient sub-cellular targeting	Measurement problem for short-lived ROS due to encapsulation Unstable and irreversible It is not specific	[122,123,124,125,126,127,128]
**Nano surface energy transfer (NSET) (e.g., gold nanoparticles)**	Detection of intracellular nM ROS	Stability (PH Dependent) outside a reducing environment	It is irreversible	[129]
**Electrochemical (e.g., carbon nanotubes, graphene-modified electrodes, or metal nanoparticle-based nanoelectrodes)**	Detection nM and μM of H_2_O_2_	Fast detection It has higher sensitivity	Unusable for in-vivo models	[130,131,132,133,134,135,136,137]

**Table 4:** Comparative evaluation of commonly used models and methods for oxidative stress research in cardiovascular diseases.

Model/Method	Reliability	Reproducibility	Translational Relevance	Best Use Case
**H_2_O_2_/TBHP (in vitro)**	High	High	Low	Mechanistic studies of ROS signaling
**H9c2 cell line**	High	High	Low–Moderate	Screening and pathway analysis
**Primary cardiomyocytes**	Moderate	Moderate	Higher than cell lines	Physiological validation
**3D/microfluidic systems**	Moderate	Low–Moderate	Moderate–High	Tissue-level interactions
**ISO-induced model**	High	Moderate–High	Moderate	Drug screening, acute injury
**DOX-induced model**	High	Moderate	Moderate	Cardiotoxicity studies
**HFD model**	Moderate	Low–Moderate	High	Metabolic and chronic disease studies
**Hypoxia/Reoxygenation**	Moderate	Moderate	High	Ischemia–reperfusion research
**DCFDA assay**	Moderate	High	Low	High-throughput ROS screening
**EPR/ESR**	High	High	High	Direct ROS detection (gold standard)
**Seahorse assay**	High	Moderate	High	Mitochondrial function analysis

## Data Availability

No new data were created or analyzed in this study.
